# Phosphoinositide-Dependent Signaling in Cancer: A Focus on Phospholipase C Isozymes

**DOI:** 10.3390/ijms21072581

**Published:** 2020-04-08

**Authors:** Eric Owusu Obeng, Isabella Rusciano, Maria Vittoria Marvi, Antonietta Fazio, Stefano Ratti, Matilde Yung Follo, Jie Xian, Lucia Manzoli, Anna Maria Billi, Sara Mongiorgi, Giulia Ramazzotti, Lucio Cocco

**Affiliations:** Cellular Signalling Laboratory, Department of Biomedical and Neuromotor Sciences (DIBINEM), University of Bologna, Via Irnerio 48, 40126 Bologna, Italy; eric.owusuobeng2@unibo.it (E.O.O.); isabella.rusciano3@unibo.it (I.R.); mariavittoria.marvi2@unibo.it (M.V.M.); antonietta.fazio2@unibo.it (A.F.); stefano.ratti@unibo.it (S.R.); matilde.follo@unibo.it (M.Y.F.); jie.xian2@unibo.it (J.X.); lucia.manzoli@unibo.it (L.M.); annamaria.billi@unibo.it (A.M.B.); lucio.cocco@unibo.it (L.C.)

**Keywords:** phosphoinositides, phospholipase C, cancer, phosphatidylinositol

## Abstract

Phosphoinositides (PI) form just a minor portion of the total phospholipid content in cells but are significantly involved in cancer development and progression. In several cancer types, phosphatidylinositol 3,4,5-trisphosphate [PtdIns(3,4,5)P_3_] and phosphatidylinositol 4,5-bisphosphate [PtdIns(4,5)P_2_] play significant roles in regulating survival, proliferation, invasion, and growth of cancer cells. Phosphoinositide-specific phospholipase C (PLC) catalyze the generation of the essential second messengers diacylglycerol (DAG) and inositol 1,4,5 trisphosphate (InsP_3_) by hydrolyzing PtdIns(4,5)P_2_. DAG and InsP_3_ regulate Protein Kinase C (PKC) activation and the release of calcium ions (Ca^2+^) into the cytosol, respectively. This event leads to the control of several important biological processes implicated in cancer. PLCs have been extensively studied in cancer but their regulatory roles in the oncogenic process are not fully understood. This review aims to provide up-to-date knowledge on the involvement of PLCs in cancer. We focus specifically on PLCβ, PLCγ, PLCδ, and PLCε isoforms due to the numerous evidence of their involvement in various cancer types.

## 1. Introduction

Lipids, most importantly phospholipids, are the main structural constituents of all cellular membranes. Phospholipids include phosphatidylethanolamine, phosphatidylglycerol, phosphatidylserine, phosphatidylcholine, phosphoinositides, phosphatidic acid and sphingomyelin [1,2]. Using magnetic resonance spectroscopy technologies to study various cancer types, it was established that phospholipids, such as phosphatidylcholine and phosphatidylethanolamine contents were aberrant in cancer [3]. In addition, alterations in phospholipid levels were seen in human breast cancer tissues with respect to healthy control tissues [4].

**Figure 1:**
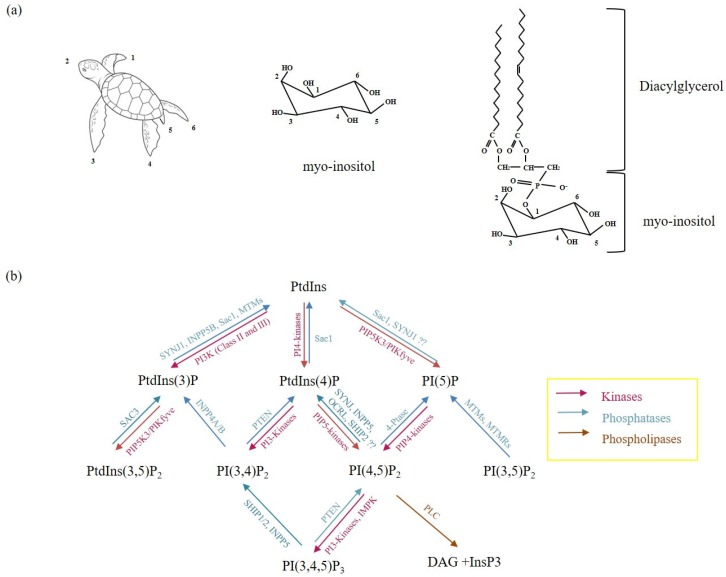
Phosphoinositides and their metabolic enzymes in the Phosphoinositide (PI) cycle. (**a**): Schematic diagram showing the positions of the individual hydroxyl groups in myo–inositol following Agranoff’s turtle concept. (**b**): metabolism of phosphoinositides depends on several lipases, phosphatases and kinases to catalyze PI-dependent reactions. ?? represents enzymatic reactions that are not completely understood.

Phosphoinositides (PIs) are the most studied phospholipids. They include the precursor phosphatidylinositol (PtdIns) and their phosphorylated derivatives, polyphosphoinositides (PPI). PtdIns has a diacylglycerol (DAG) backbone that is phosphodiesterified to a six-carbon cyclic polyol, myo-inositol (CHOH)_6_ head group. The myo–inositol head group of PtdIns is composed of one axial and 5 equatorial hydroxyl groups assuming a turtle conformation [5,6]. PtdIns can be phosphorylated on three out of five equatorial hydroxyl groups at positions -3,-4,-5 on the inositol ring to yield the seven different types of PPIs: PtdIns(3,4,5)P_3,_ PtdIns(4,5)P_2_, PtdIns(3,5)P_2_, PtdIns(3,4)P_2_, PtdIns3P, PtdIns4P and PtdIns5P (Figure 1) [5].

PIs form just a minor fraction of the total phospholipid content in eukaryotic cell membranes. However, they function in diverse roles, ranging from regulating essential biological processes such as cell adhesion [7], migration [8], apoptosis [9], vesicular trafficking [10], to post-translational modifications [11]. All these cellular processes are consistent with the hallmarks of cancer. Furthermore, PI levels are regulated by several lipid kinases, phosphatases and phospholipases in response to different external stimuli, with a plethora of studies showing that a deregulation of the PI metabolism mediated by these enzymes is implicated in several diseases [12,13,14].

All these evidences highlight the importance of understanding how PIs and their metabolic enzymes mediate critical roles implicated in cancer. This review sums up the current knowledge on the involvement of PI metabolic enzymes phospholipase C (PLC) in various cancer types. We have described the structure and activation of the various PLC isoforms and their influence in cell proliferation, survival, tumor growth, as well as in cell migration, invasiveness, and metastasis. However, to the best of our knowledge, there is currently no published evidence of the direct roles of PLCζ and PLCη in cancer. As such we focus on PLCβ, PLCγ, PLCδ, and PLCε. On the other hand, readers can also find in-depth information on the other phospholipases, i.e., PLA, PLB and PLD [15,16,17].

## 2. Phospholipases

Phospholipases are a family of phospholipid metabolizing enzymes that catalyze the breakdown of phospholipids into biologically active lipid mediators which control several physiological cell functions [18,19,20]. Currently, four major families of phospholipases have been identified: phospholipases A, B, C, and D (PLA, PLB, PLC, and PLD, respectively). They are based on the type of reaction they catalyze. For example, the isoforms of PLA produce free fatty acids and lysophospholipids by targeting the glycerol component of phospholipids whereas PLD hydrolyses phosphatidylcholine to generate choline and phosphatidic acid. Most studies on phospholipases are focused on their sub-families. In fact, each family member has specific targets and functions, but it shares common signaling pathways with other sub-family members [15,20]. PLCs have been studied extensively in various cancer types. However, understanding their regulatory roles in the oncogenic process and the potential crosstalk among distinct PLCs remain enigmatic.

PLCs comprise of 6 sub-family members which hydrolyze PtdIns(4,5)P_2_ to generate the two essential intracellular second messengers diacylglycerol (DAG) and inositol 1,4,5 trisphosphate (InsP_3_) following their activation. This promotes the activation of protein kinase C (PKC) and the release of calcium ions (Ca^2+^) from the intracellular stores, respectively [21,22,23,24]. InsP_3_ detaches from the membrane and interacts with InsP_3_-specific receptors to regulate Ca^2+^ release, while DAG remains membrane-bound to mediate the activation of PKC upon Ca^2+^ release [25]. This represents a key point in cell signaling in various cancer types. For instance, PKC is reported to be involved in cell proliferation, differentiation, migration and growth [26] while Ca^2+^ release is critical in the regulation of cancer cell motility, division and death [27,28]. Simultaneously, the decrease of PtdIns(4,5)P_2_ concentration, via its hydrolysis by PLCs, has been reported to also yield several essential signaling cascades implicated in cancer, especially cell migration. This is due to the influence of PtdIns(4,5)P_2_ activity in the modulation of various pleckstrin homology (PH) domain-containing proteins, as well as actin regulatory proteins [23,29].

### 2.1. Structure and Activation of PLCs Implicated in Cancer

**Figure 2:**
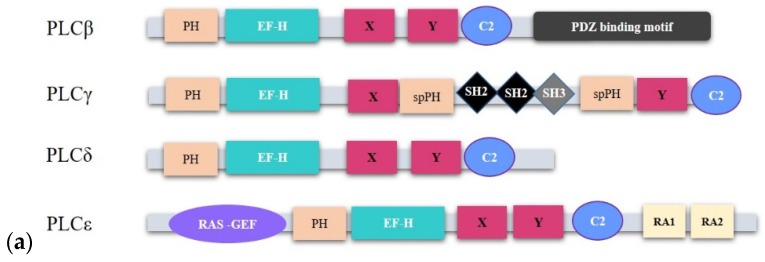

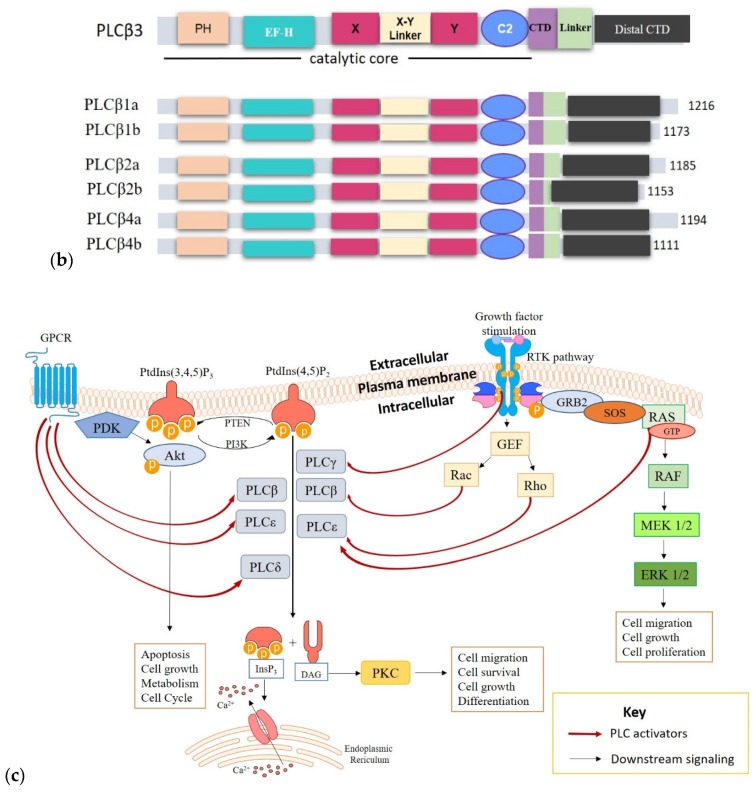
The structure and activation of phospholipase C (PLC) isozymes. (**a**): cartoon representing the structural components of PLC family members implicated in cancer. PLC isoforms possess structurally conserved domains such as the EF, X and Y catalytic core, and the C2 domains. However, the organization of their regulatory domains follows a subtype dependent manner. (**b**): PLCβ isoforms and their spliced variants show conserved structural features with minor differences at the C-terminal domain (CTD). They incorporate a core set of domains consisting of an N-terminal PH domain, four EF-hand motifs, a X–Y catalytic site, a C2 domain, and a C-terminal domain with a linker between the proximal and distal ends. The various isoforms possess varied lengths and sequences occurring within the C-terminal extensions. (**c**): PLCs are activated by different stimuli to mediate the hydrolysis of PtdIns(4,5)P_2_ into the second messengers InsP_3_ and DAG, which subsequently promote the intracellular release of Ca^2+^ from the endoplasmic reticulum (ER) and activation of PKC, respectively. PKC activation following DAG and Ca^2+^ release promotes cell migration, cell survival and differentiation. Some PLCs are activated by more than one mechanism. For example, PLCε can be activated by the rat sarcoma (RAS) protein and the RAS homolog family member (Rho) as well as G-proteins. PLCβ, especially PLCβ2 can be activated via the classical G-protein pathway but also through RAS-related C3 botulinum toxin substrate (Rac) GTPases. However, the routes of activation may be cell type-specific or dependent on stimuli. Red-colored arrows represent reactions that activate PLCs while black arrows show downstream signaling paths.

PLCs are composed of 13 distinct isozymes grouped into six sub-families: PLCβ (1,2,3 and 4), PLCγ (1 and 2), PLCδ (1,3 and 4), PLCε, PLCη (1 and 2), and PLCζ based on their structural similarities and organization [5,25,30]. So far, all reported PLC isoforms bear conserved regions such as the X and Y regions, which make up the catalytic domain, the PH domain, the EF-hand (EF-H) domain and the PKC homology (C2) domain. Each of these domains have some functional roles. For example, the PH domain binds to PtdIns(4,5)P_2_ with high affinity and specificity, the EF-H domain play scaffolding roles to support guanosine triphosphate (GTP) hydrolysis upon G-protein coupled receptor (GPCR) binding, and the C2 domain participates in intra- and inter-molecular signaling processes [23,25]. Regulatory domains such as the C2 domain, the EF-H motif, RAS associating (RA) domain and the PH domain are uniquely distributed in PLC subtypes (Figure 2a). This may explain their distinct functions and/or tissue distribution [13]. PLCs are distributed across several cellular compartments depending on the localization of their substrate PIs. For instance, PIs have been shown to localize within the nucleus together with their metabolic enzymes which help to generate a separate PI metabolism that is distinct from the cytoplasmic PI cycle [21,31]. Some PLC isoforms like PLCβ1 [32,33], PLCγ1 [34], PLCδ1 [35], PLCδ4 [36], and PLCε [37] have been reported to localize in the nucleus. Essential to this review, there are reports of the implications of nuclear PLC signaling in cancer [12,38,39].

#### 2.1.1. PLCβ

The PLCβ subfamily is one of the most studied PLCs and it comprises of 4 isoforms: PLCβ1, PLCβ2, PLCβ3, and PLCβ4 [13] with some existing as spliced variants. This is the case of PLCβ1: a and b, PLCβ2: a and b and PLCβ4: a and b. Even though PLCβ isoforms exhibit conserved structural features, some variations have been identified. The length of each isoform may be different, and this is due to the variations in the length and sequence of the C terminal extension (Figure 2b) [25]. PLCβ isoforms are activated by the Gαq- and Gβγ subunits of heterotrimeric G proteins [13]. However, Rac which is one of the Rho family of GTPases has been reported to also activate PLCβ2 due to its high affinity binding to the PH domain of PLCβ2 [40] (Figure 2c). PLCβ1 is the most studied PLCβ isoform expressed in the nervous system, particularly in the cerebral cortex and hippocampus [41], as well as in the cardiovascular system [42,43]. On the other hand, PLCβ2 isoforms are expressed in hematopoietic cells and platelets, where they regulate chemotaxis [25,44]. PLCβ3 is expressed in the liver, brain, hematopoietic cells, the cardiovascular system, and the parotid gland, where it mediates proliferation and chemotaxis [25]. Finally, PLCβ4 is expressed in the cerebellum and in the retina for visual processing events after phototransduction [45].

#### 2.1.2. PLCγ

PLCγ is composed of 2 isoforms, PLCγ1 and PLCγ2. PLCγ1 is ubiquitously expressed, as compared to PLCγ2, which is mainly restricted to hematopoietic lineages [46]. PLCγ isozymes act through phosphorylation mediated by the binding of their SH2 domain to phosphorylated tyrosine residues of activated and non-activated receptor tyrosine kinases (RTKs) (Figure 2c). Most of the tyrosine kinases implicated in the activation of PLCγ are members of the growth factor receptor superfamily, which includes platelet-derived growth factor (PDGF), fibroblast growth factor (FGF), hepatocyte growth factor (HGF), epidermal growth factor (EGF), insulin-like growth factor (IGF) and vascular endothelial growth factor (VEGF). However, in some cells, such as platelets, PLCγ activation depends on phosphoinositide 3-kinase (PI3K) [46,47,48]. Additionally, PLCγ was demonstrated to be activated via phosphorylation of tyrosine 1253 and tyrosine 783 residues in the nuclei of cells from early breast cancer patients, indicating a role of PLCγ in the nuclear PI cycle [47].

#### 2.1.3. PLCδ

The PLCδ sub-family is composed of three different isoforms: PLCδ1, with variants 1a and 1b; PLCδ3, and PLCδ4, with variants 4a, 4b, and 4c. Compared to PLCβ and PLCγ, PLCδ is relatively a smaller isozyme [49]. PLCδ enzymes, and more specifically PLCδ1, are activated via GPCR-mediated calcium mobilization (Figure 2c). The various isoforms of PLCδ are distributed across multiple cellular sites. It has been identified in the nucleus and it possesses both nuclear export and import sequences that contribute to its shuttling between the cytoplasm and nucleus [35,50]. PLCδ1 is localized mainly within the cytoplasm, PLCδ3 in membrane fractions and PLCδ4 mainly in the nucleus [49]. PLCδ plays several essential roles in different tissue types. In particular, PLCδ1 is important for skin homeostasis and for tissue metabolism, while PLCδ3 promotes microvilli formation in enterocytes and the radial migration of neurons in the cerebral cortex during brain growth [13,51].

#### 2.1.4. PLCε

PLC210, the first PLCε homologue, was discovered by Kataoka and colleagues in 1998, when they were screening LET-60 RAS effectors in *C. elegans* [52]. Further studies revealed two spliced variants of PLCε1 (PLCε1a and PLCε1b). They differ at the amino terminus, are tissue-specific and vary in size by 25kDa [53]. However, the unique roles played by these two isoforms have not been established yet. Compared to the other PLCs, PLCε possesses the largest molecular size, being about 230 kDa. PLCε is activated via several unique signaling pathways. Lopez and colleagues demonstrated that PLCε was not activated by Gαq, but by Gα_12_ and Gβγ subunits of G-proteins [54]. Furthermore, RhoA [55] and the RAS family of small GTPases, (RAS, RAS-related protein (Rap1) and Rap2B) [29,56] are able to activate PLCε through direct binding to the various domains of PLCε (Figure 2c). PLCε localizes within different cellular sub-compartments following its binding to Rap1 and RAS [29] or different cellular stimulations [37]: in perinuclear space [29,57], cytoplasm, and plasma membrane [29]. The ability of PLCε to be activated via several stimuli and its distribution across several cellular sub-compartments makes it one of the most complex signaling hubs. PLCε signaling serves as a converging point that links the RTK signaling pathway to the PLC pathway, by sensing and mediating communication between both pathways [53].

## 3. PLCs in Cancer Development and Progression

Due to the involvement of PLCs in several cellular signaling pathways, alterations in the activity and expression of the various isoforms of PLC have been detected in different human cancer types. For example, PLCβ in neuroendocrine tumors and hematopoietic malignancies [58,59,60,61], PLCγ in breast cancer, colon carcinoma [62,63], lymphocytic leukemia and angiosarcoma [64], PLCδ in esophageal squamous cell carcinoma (ESCC) [65], and PLCε in gastric cancer [66] and colorectal cancer [67]. Nevertheless, the regulatory roles played by PLCs in cancer remain elusive and sometimes controversial. For example, PLCε is upregulated in gastric cancer [66], but downregulated in colorectal cancer [67]. In addition, PLCε knockout mice, generated by two different groups, showed contrasting oncogenic roles played by PLCε: one being pro-oncogenic [68] and the other one being anti-oncogenic [69]. Recent studies have reported PLCδ1 as a tumor suppressor in breast cancer [70] and ESCC [65]. Even as all these reports support the fact that PLCs are involved in cancer, very little has been done at the clinical level. So far, small molecules that target PLCs like U73122, hispidospermidine, Vinaxanthone, CRM-51005, CRM-51006 and caloporoside are in preclinical testing [71]. Interestingly some of these have been shown to promote anti-tumor activities [72,73].

In cancer, alterations in cell growth, proliferation, survival and cell migration are central in the development and progression of the disease. PLCs have been demonstrated to be involved in these essential processes in cancer [70,74,75].

### 3.1. PLCs in Cancer Cell Proliferation, Survival and Tumor Growth

**Figure 3:**
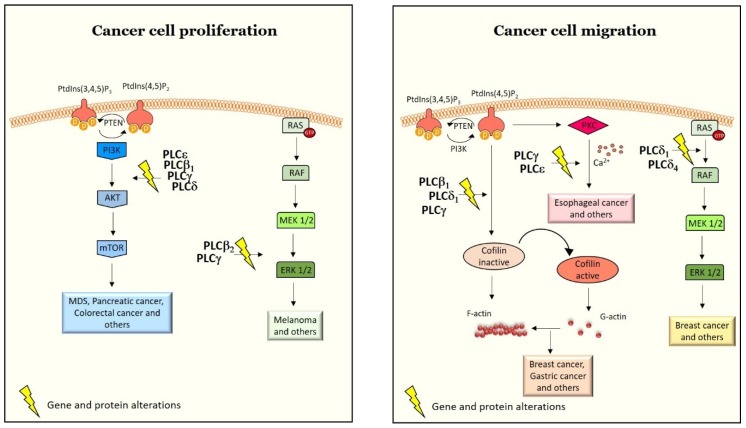
Cartoon representation of PLC-mediated cell proliferation and migration in different cancer types. Currently, except PLCζ and PLCη, all other PLCs have been shown to be directly involved in the regulation of several cellular processes in cancer such as, cell proliferation and cell migration. Alterations in the expression of PLC isoforms at both genetic and protein levels in different cancer types have been shown to affect essential pathways such as the PI3K/Akt/mTOR and the RAS/RAF/MAPK/ERK pathways implicated in cancer cell survival, growth and proliferation. Similarly, these alterations also affect essential mechanisms like actin reorganization via the activation of cofilin, implicated in regulating cell migration. In addition, molecular alterations in PLCδ expression controls cell migration in breast cancer via ERK signaling. The yellow lightning symbol represents molecular alterations in gene or protein expression, that is, either upregulation or downregulation of the specified PLC isoforms.

Activated PLC isozymes are interconnected with several pathways, such as the PI3K/protein kinase B (PKB/Akt)/mammalian target of rapamycin (mTOR) (PI3K/Akt/mTOR) pathway [76], RAS/rapidly accelerated fibrosarcoma (RAF)/mitogen activated protein kinase (MAPK)/extracellular signal-related kinase (ERK) pathway [77], and the Janus kinase (JAK)/signal transducer and activator of transcription (STAT) [78] pathway that are major regulators of cell growth and proliferation in cancer cells (Figure 3). In the next paragraphs, we show how PLCs interact with these pathways to control cell growth, proliferation and survival in cancer.

Indeed, cancer cells hijack the PI3K/Akt/mTOR pathway to promote the induction of survival signals, inhibit apoptotic or cell death signals and acquisition of chemoresistant phenotypes [79,80]. As such, several cancer therapies target this pathway to inhibit the cell survival effects of cancer cells. So far, over fifty PI3K/Akt/mTOR inhibitors have been generated and are in different stages of development, with an increasing number reaching clinical trials. For instance, analogs of rapamycin, everolimus and temsirolimus which are inhibitors of mTOR complex 1(mTORC1) are clinically approved for treating several cancer types like advanced renal cell carcinoma, pancreatic neuroendocrine tumors, and advanced breast cancer. Notably, they reduce cell proliferation in these cancer types [81]. CAL-101 or idelalisib which is an approved PI3K inhibitor induces elevated cell death in chronic lymphocytic leukemia [82]. Interestingly, alterations in both PLCβ1 and PI3K/Akt/mTOR pathways have been associated with myelodysplastic syndromes (MDS) and acute myeloid leukemia (AML) [57,83,84]. MDS are a group of hematological diseases characterized by impairment in cell differentiation and proliferation [79,85]. About 30% of confirmed MDS cases evolve into AML, so it is imperative to identify biomolecular markers associated with the risk of AML evolution in MDS patients [86,87,88]. Our group showed that monoallelic deletion of PLCβ1 gene in MDS patients showed worse clinical outcomes with an increased probability of evolving into AML [89]. To understand this, we further demonstrated that PLCβ1 mRNA expression can act as a dynamic marker to detect the effectiveness of the DNA methyltransferase inhibitor, azacitidine, which is commonly used to treat MDS patients to delay AML evolution. It was shown that, increase in PLCβ1 mRNA expression correlates with positive clinical outcomes while PLCβ1 decrease is associated with worse clinical outcomes in MDS patients [90]. Of particular note, PLCβ1 expression in MDS patient-derived cells inversely correlates with Akt phosphorylation. Samples from MDS patients showing positive clinical outcomes expressed low levels of phosphorylated Akt [84]. This may indicate that alterations in PLCβ1 expression and Akt activation in MDS may deregulate the cell cycle of MDS cells, resulting in the inhibition of apoptotic mechanisms and promotion of cell survival of MDS cells [83,91,92].

On the other hand, upstream activators of PLCγ, like EGF and PDGF are major regulators of cell growth and proliferation [93]. PDGF has been shown to activate mTOR and Akt but the mechanism behind this is not completely understood. Interestingly, constitutively active PI3K promotes PLCγ activation [94]. Using a dominant negative PLCγ mutant and PLCγ inhibitors in NIH-3T3 cells resulted in the inhibition of Akt phosphorylation on serine 473 but not on threonine 308. This observation may be explained by an interesting report showing an interaction between PLCγ and Pyruvate dehydrogenase kinase 1 (PDK1) which is a promoter of Akt activation upon EGF stimulation. PDK1 was indeed shown to participate in the activation of PLCγ in a partially dependent manner [95]. Furthermore, Rictor, which is a subunit of the mTORC2 complex, promoted PDGF-mediated phosphorylation of Akt on serine 473, along with PLCγ phosphorylation [95]. In addition, the nuclear PLCγ content was demonstrated to be involved also in the activation of nuclear PI3K as well as regulating the expression of cell cycle proteins like cyclin-dependent kinase 4 (CDK4) and cyclin D1 (CCND1) [47]. Furthermore, functional studies in ESCC cell and animal models revealed that PLCδ1 is able to suppress the tumorigenic ability of ESCC cells in both in vitro and in vivo studies [65]. In this study, PLCδ1 expression was absent in 52% of primary ESCCs and 4 out of 9 ESCC cell lines, this significantly corresponded to promoter hypermethylation and copy number loss. The anti-tumor roles played by PLCδ1 in ESCC included the induction of a cell cycle arrest at the G1/S phase via the upregulation of p21 and the downregulation of Akt phosphorylation. On the contrary, Wang et al. showed that downregulation of PLCε inhibited cell proliferation in pancreatic cancer cell lines via the PTEN/Akt pathway and thus, PLCε was acting as an oncogene [76].

In addition, a tissue microarray analysis of 77 breast cancer tumor samples showed that PLCβ2 is highly expressed in breast cancer and is associated with a poor clinical outcome. PLCβ2 expression correlated with tumor size, proliferation index and tumor grade [96]. In line with this microarray study is a recent functional study reporting that the knockdown of PLCβ2 expression in melanoma cells negatively affects cell viability and promotes cell apoptosis by altering p53 and pro-apoptotic proteins, caspase 3 and Bcl-2-associated X (Bax). Interestingly, PLCβ2 mediated these responses by activating the RAS/RAF/MAPK/ERK pathway [77]. This pathway remains one of the promising therapeutic targets in cancer therapies due to its influential roles in cell proliferation, differentiation, apoptosis, growth and survival [97,98]. Lastly, the authors showed that PLCβ2 regulated the transition of cells from the G0/G1 phase to the G2/M phase, without altering the cell cycle proteins [77]. Thus, PLCβ2 may promote G2/M progression of melanoma cells, an essential event in cancer evolution. Many studies have also reported an interaction between PLCγ and the MAPK signaling cascade [99,100]. For instance, NGF-mediated activation of the RAS/RAF/MAPK/ERK pathway in PC12 pheochromocytoma cells was dependent on PLCγ activity [99]. Moreover, Colin et al. showed that PLCγ can also be a MAPK substrate, through PLCγ1 interaction with ERK2 and phospho-ERK2 in both rat brain and in vitro studies [100].

Accumulating evidence suggests that PLCs can regulate cell survival and proliferation in cancer cells through the JAK/STAT pathway [78]. For instance, the SH2 domain of PLCγ can interact with the tyrosine 705 residue of STAT3, resulting in PLCγ activation. This mediates PLCγ-associated cancer mechanisms. In colorectal cancer, the crosstalk between PLCγ and phosphorylated STAT3 may play a critical role in tumorigenesis [101]. As for other PLCs, in a mutant mice model, Xiao and colleagues demonstrated that the loss of PLCβ3 can promote tumor development and growth [78]. 50% of PLCβ3−/− mutant mice (*n* = 36) died within 16 months after genetic manipulation, as compared to 100% survival of wildtype mice. The death of the PLCβ3−/−mutant mice was attributed to the development of various tumors, including myeloproliferative disease and lymphoma. The authors suggested that PLCβ3 knockout led to an increase of hematopoietic stem cells (HSCs) and a decrease of STAT5, although the full mechanism of action is unknown. This induced elevated cell proliferation, survival and myeloid differentiation [78].

### 3.2. PLCs in Cell Migration, Invasiveness and Metastasis

The majority of cancer-related deaths are not due to primary tumor growth, but to the ability of primary tumors to invade new sites away from original tumors. During invasion, single tumor or tumor cell clusters detach from primary tumors, assume motile functions and migrate to new sites through their surrounding extracellular matrix. A study on metastatic breast cancer cell lines established that PLCβ1 interacts with the Protein Tyrosine Phosphatase Receptor Type N2 (PTPRN2) protein. Moreover, both proteins were highly expressed and correlated with metastatic relapse in humans [59]. The authors showed that PLCβ1 and PTPRN2 control cell migration by enzymatically inducing a reduction in PtdIns(4,5)P_2_ levels at the plasma membrane [59]. PtdIns(4,5)P_2_, which is a substrate of PLCβ, has been reported to regulate actin dynamics to promote cell migration [102,103]. Reduction of PtdIns(4,5)P_2_ levels promotes the release of the actin-binding factor, cofilin, into the cytoplasm, where it becomes active and modulates actin turnover dynamics to enhance cell migration and metastasis [59,104]. Furthermore, PLCβ2 expression correlates with the clinical outcome of breast cancer patients and in fact, PLCβ2 promotes migration and invasiveness in human breast cancer-derived cells [96]. In this study, PLCβ2 mediated hydrolysis of PtdIns(4,5)P_2_ caused an increase in the expression of the cytoskeletal component actin as PtdIns(4,5)P_2_ levels decreased. This suggests that PLCβ2 may regulate cell motility and invasion in breast cancer cell lines by altering PtdIns(4,5)P_2_ expression levels [96,104]. Similarly, PLCγ1-mediated hydrolysis of PtdIns(4,5)P_2_ induces the release of actin-modifying proteins such as gelsolin, which promotes actin reorganization behind the leading edge. Gelsolin also works in conjunction with other actin-binding proteins, like profilin and cofilin, to regulate Arp2/3 branching at the leading edge [46,105,106]. Importantly, gelsolin is bound to PtdIns(4,5)P_2_ in its resting state and becomes active in cell migration only after PtdIns(4,5)P_2_ hydrolysis by PLCγ1 [105]. On the other hand, PLCδ1 expression is downregulated in about 84% of gastric cancer cell lines and this correlates with PLCδ1 promoter methylation. Ectopic expression of PLCδ1 in PLCδ1 downregulated gastric cancer cells significantly inhibits cell migration. In PLCδ1 transfected cells, PLCδ1 expression may suppress migration by blocking cytoskeletal reorganization through cofilin inactivation and also inducing the downregulation of matrix metalloproteinase 7 (MMP7) [107].

Another study by Hatziapostolou et al. showed that the mitogen-activated protein 3-kinase (MAPKKK/MAP3K) tumor progression locus 2 (Tpl2) promotes cell migration via a PLCβ3-dependent pathway [27]. Using pharmacological and genetic tools on both in-vitro and in-vivo samples, it was shown that Tpl2 promotes the release of Ca^2+^ into the cytosol through PLCβ3 activation. In addition, Tpl2 also activates ERK by phosphorylating mitogen-activated kinase (MEK). The authors demonstrated that the PLCβ3-Ca^2+^ and MEK-ERK pathways crosstalk to control cell migration in MDA MB 231 breast cancer cells. More importantly, the promotion of cell migration by Tpl2 was dependent on both pathways. PLC-mediated Ca^2+^ influx has been demonstrated to promote the disassembly of focal adhesions at the rear of migrating cells and assembly of focal adhesions at the leading edge, an essential event in migrating cells [27,108]. In support of this study, there is mounting evidence of the ability of the downstream effectors of PLCγ, PKC and Ca^2+^ to support cell migration, by regulating cell adhesion turnover and acto–myosin contractility [46]. Interestingly, reducing PLCγ expression by molecular tools inhibited metastasis in nude mice [48]. Moreover, U73122, which is a potent inhibitor of PLCγ activity, blocked glioma cell motility and invasion in fetal rat brain aggregates [72]. In addition, metastasis was reduced in an in vivo prostate carcinoma model expressing a dominant negative fragment of PLCγ [109], with similar observations in an in vitro model of the neck and squamous cell carcinoma (HNSCC) upon PLCγ inhibition [110]. Regarding the use of inhibitors, Hiroshi and colleagues also decided to investigate the effects on cell migration in HNSCC cells following the use of a combinatorial inhibition approach to inhibit both PLCγ1 and the protooncogene c-Src which is a nonreceptor tyrosine kinase [73]. U73122 was used to inhibit PLCγ1 whereas the Src family inhibitor AZD0530 was used to inhibit c-Src. Both PLCγ1 and c-Src are linked to EGFR. EGFR activates PLCγ1 while c-Src interacts directly with EGFR in HNSCC cells upon EGF stimulation, hence, it was interesting to study the combinatorial role of PLCγ1 and c-Src in HNSCC. The authors showed that a combined inhibition of PLCγ1 and c-Src in HNSCC cells resulted in an increased abrogation of EGF-induced cell invasion compared to using each inhibitor alone [73]. This supports the fact that PLCγ1 and c-Src interact upon EGF stimulation and that activation of both targets via EGFR promotes invasion in HNSCC cells.

PLCε mediated activation of the RAS-MAPK pathway promotes cancer cell invasion or metastasis by phosphorylating serine 68 of Twist1 proteins. Twist1 is a transcriptional factor that regulates epithelial-to-mesenchymal transition and cell motility [54,111]. Interestingly, Fan et al. reported that knocking down PLCε in prostate cancer cells significantly reduces the phosphorylation levels of Twist1 proteins through MAPK signaling. Moreover, they showed that cell migration or tumorigenesis was suppressed in PLCε knockdown cells as well as tumor xenografts [112]. A recent study by Yongzhu and Chunyan revealed a higher expression of PLCε in esophageal cancer cell lines, and this positively correlated with increased expression of PKCα [113]. The authors downregulated PLCε expression with PLCε siRNA in esophageal cancer cells and for the first time, they demonstrated that PLCε reduction in esophageal cancer cell lines inhibited cell migration and invasion through PLCε mediated downregulation of PKCα and Nuclear Factor kappa-light-chain-enhancer of activated B cells (NF-κB) [113]. Consistent with these observations is the study by Marta and colleagues on fibroblasts derived from transgenic animals with null or mutated alleles where null fibroblasts showed impaired chemotaxis towards PDGF [114]. Moreover, they reported that PLCε controls cell chemotaxis via its PLC activity instead of its RAS-GEF activity [114]. However, this study failed to confirm that PKC regulated chemotaxis, but, given that PKC is downstream of PLC activity, PLCε-mediated PKC activation may be involved in chemotaxis. Some growth factor receptors known to activate PLCγ are also implicated in conferring invasive advantages to several carcinomas in order to promote tumor progression [46,105,110]. These growth factors crosstalk frequently to drive tumor progression. In glioblastoma, PDGFR, NGF, IGF-1 and, most essentially EGFR upregulation, mediates the migration of glioblastoma cells into normal brain tissues. However, inhibiting PLCγ disrupts the invasion of glioblastoma cells into normal brain tissues, regardless of the combined signaling effects of PDGFR, NGF, IGF-1, and EGFR upregulation [72,93]. In MDA-MB-231 breast carcinoma cells, PLCγ downregulation decreased membrane ruffle formation, by inhibiting both EGF and serum-induced activation of the small GTP binding protein Rac1, a well-established protein crucial in the generation of actin polymers at the leading edge of migrating cells [48].

The tumor suppressor roles played PLCδ1 in ESCC does not only end in inhibiting cell growth but also their ability to inhibit cell migration. Fu and colleagues demonstrated from their tissue microarray studies that PLCδ1 downregulation correlates significantly with metastasis in ESCC [65]. As such, the authors went on to study the functional role of PLCδ1 in ESCC metastasis. Exogenous expression of PLCδ1 in ESCC cell lines increased cell adhesion on collagen as well as decreasing cell migration when compared to control cells, suggesting that PLCδ1 may be involved in cell migration. However, the mechanism by which PLCδ1 mediated this function in ESCC was not studied [65]. The study by Shao et al. showed that PLCδ1 expression was downregulated in breast cancer and that, PLCδ1 activity inhibited breast cancer cell migration via the inhibition of the ERK1/2/β-catenin/MMP pathway (Table 1). Most importantly, this activity was dependent on Kinesin-Like Protein KIF3A (KIF3A) signaling [70]. Indeed, KIF3A is a downstream effector of PLCδ1 and its expression is inversely correlated to PLCδ1 expression. Therefore, down-regulation of KIF3A expression, which corresponds to PLCδ1 increase, inhibited cell migration and invasion by blocking the ERK1/2/β-catenin/MMP pathway. Conversely, knocking down PLCδ1 reactivates the ERK1/2/β-catenin/MMP pathway and induces cell migration [70]. Additionally, overexpression of PLCδ4 in MCF-7 breast carcinoma cell line promotes oncogenic properties through the upregulation of Erb-B2 Receptor Tyrosine Kinase 2 (ErbB2) expression and activation of the ERK pathway [115] which is implicated in cell migration through its regulation of focal adhesion proteins, paxillin and focal adhesion kinase (FAK). Focal adhesion turnover promotes cell retraction which is essential for cell migration [116].

## 4. Conclusions

This review shows that PLCs play a central role in the regulation of cellular signaling in cancer mechanisms. So far, small molecules that target PLCs are still in preclinical testing even though some promote anti-tumor activities. The ability of the various PLC isoforms to alter the signaling of major oncogenic pathways implicated in cancer like the PI3K/Akt/mTOR, RAS/RAF/MAPK/ERK, and JAK/STAT pathways underscore why it is important that future studies focus on understanding the therapeutic potential of PLCs in cancer. Substantial progress has been made so far in understanding the specific functions and signaling events associated with PLCs, however, there is still a lot to understand. For instance, it will be interesting to clear the controversies surrounding the roles of some PLCs in cancer, either as a tumor suppressor or as a tumor promoter. For example, PLCε can play both pro and anti-tumor roles in cancer. Since all identified PLCs regulate intracellular Ca^2+^ release, understanding whether this process is regulated by a combinatorial function of all PLCs will be essential. In fact, there are still some open questions that need addressing, like, are there compensatory mechanisms that preserve the functions of PLCs should one or more PLCs be downregulated or how does PLCs affect each other in cancer? A challenge for future studies will be to fully demystify the role of the diverse signaling pathways mediated by the individual PLC family members, as well as possible crosstalk among PLCs in the regulation of cellular functions implicated in cancer. These are some of the most essential questions that still need to be answered to pave way for a better comprehension of PLCε in cancer development and progression. The involvement of PLCs in several cancer types makes this field of research highly essential in medicine, as increasing our understanding of PLCs may generate novel therapeutic targets for pharmacological interventions.

## Figures and Tables

**Table 1:** Major pathways altered by PLCs in various cancer types.

PLC Isoforms	Tumor Entity	Tumor Specificity	Mode of Experimentation	Pathways Altered
*PLCβ1*	MDS	Diseased patients	Expression profiling	Akt/mTOR
*PLCβ2*	Melanoma	Melanoma cells	Functional studies	RAS/RAF/MAPK
*PLCβ3*	Lymphoma	Mutant mice	Functional studies	JAK/STAT
*PLCβ3*	Breast cancer	MDA-MB231 cells	Functional studies	MEK/ERK
*PLCγ*	Pheochromocytoma	PC12 cells	Functional studies	PI3K/Akt/mTOR and RAF/MEK/MAPK
	Colorectal cancer		Functional studies	JAK/STAT
*PLCδ1*	ESCC	ESCC cell lines	Functional studies	PI3K/Akt
	Breast Cancer	Diseased cell lines	Functional studies	ERK1/2/β-catenin/MMP
*PLCε*	Pancreatic cancer	Diseased cell lines	Functional studies	PTEN/Akt
	Prostate cancer	Diseased cell lines	Functional studies	RAS/RAF/MAPK

## Abbreviations

SHIP	Src homology 2 (SH2) domain containing inositol phosphatase
4-Ptase	4-Phosphatase
IPMK	Inositol polyphosphate multikinase
PI4-Kinases	Phosphatidylinositol 4-kinases
PIPK	Phosphatidylinositol phosphate kinase
PTEN	Phosphatase and tensin homolog deleted on chromosome 10
OCRL	Oculocerebrorenal syndrome of Lowe
INPP	Inositol polyphosphate phosphatase
Sac1	Phosphoinositide phosphatase Sac1
SYNJ	Synaptojanin
MTM	Myotubularin
MTMRs	Myotubularin-related proteins
RAS-GEF	RAS guanine nucleotide exchange factor
SH2	Src homology 2 domain
SH3	Src homology 3 domain
spPH	Split PH domain
PDZ	Post-synaptic density protein Drosophila disc large tumor suppressor, and zo-1 protein
GRB2	Growth factor receptor-bound protein
SOS	Son of sevenless protein
